# Transformative global models for CKD care: case studies and strategies

**DOI:** 10.1093/ckj/sfag011

**Published:** 2026-01-13

**Authors:** James O Burton, Andrew H Frankel, Katherine Kwon, María Marqués, Gengru Jiang, Jiguang Wang, Kieran McCafferty

**Affiliations:** Department of Cardiovascular Sciences, University of Leicester, Leicester, UK; Department of Renal Medicine, Imperial College Healthcare NHS Trust, London, UK; Clinical Affairs, Panoramic Health, AZ, USA; Puerta de Hierro University Hospital, Madrid, Spain; Xinhua Hospital, School of Medicine, Shanghai Jiao Tong University, Shanghai, China; Rui Jin Hospital, Shanghai Jiao Tong University School of Medicine, Shanghai, China; Department of Renal Medicine, Barts Health NHS Trust, London, UK

**Keywords:** best practice, chronic kidney disease (CKD), guideline-directed medical therapy (GDMT), management, sodium-glucose cotransporter 2 (SGLT2) inhibitors

## Abstract

Chronic kidney disease (CKD) affects approximately 10% of adults worldwide and is associated with an elevated risk of cardiovascular disease, such as heart failure, and increased prevalence of comorbidities such as type 2 diabetes. Early CKD diagnosis and intervention are crucial to prevent progression to advanced kidney disease, which imposes a significant clinical and economic burden on health systems and patients alike. Despite the availability of global CKD management guidelines, adherence remains low, particularly with respect to the use of sodium-glucose cotransporter 2 inhibitors (SGLT2is), which offer strong cardiorenal benefits particularly when initiated in a timely manner. This gap underscores the urgent need for practical solutions to translate existing guideline recommendations into improved clinical practice across the CKD management pathway, encompassing screening, treatment initiation and referral. This manuscript highlights successful global initiatives in CKD management, presenting a ‘call-to-action’ to support healthcare systems and providers in achieving improved CKD management worldwide. By bringing together eight diverse ‘Champions of Change’ initiatives from various health systems, this paper presents innovative and transformative solutions across the entire CKD management pathway, from early diagnosis to treatment. These case studies underscore the potential of tailored, context-specific strategies for transforming CKD care. By adopting core principles such as proactive screening, risk stratification strategies, multidisciplinary collaboration, knowledge-sharing and patient-centred approaches, healthcare systems and providers can adapt these successful models to their local settings, thereby advancing global efforts to prevent CKD progression and improve patient outcomes.

## INTRODUCTION

Approximately 1 in 10 adults worldwide experience chronic kidney disease (CKD) [1]. This condition is associated with poor health outcomes, an elevated risk of cardiovascular disease, including heart failure, and increased prevalence of other comorbidities, such as diabetes and hypertension [2–4]. Undetected CKD can lead to a significant underestimation of cardiovascular risk, underscoring the importance of effective CKD screening and intervention in managing long-term conditions more widely [5]. The prevalence and burden of CKD is set to rise globally, with recent projections estimating 436.6 million cases across 31 countries and regions by 2027, constituting a 5.8% increase from 2022 [4]. The World Health Organization (WHO) projects that kidney disease will become the fifth-leading cause of premature death globally by 2050 [6]. Recognizing this as a growing priority, the WHO adopted a landmark resolution at the 78th World Health Assembly to reduce the burden of non-communicable diseases by promoting kidney health, and strengthening the prevention and management of kidney disease [6].

Beyond prevalence, the global economic burden of CKD is also predicted to increase, with one study reporting that the direct annual costs across 31 countries are expected to rise from $372.0 billion in 2022 to $406.7 billion by 2027 [4]. Analyses show that the costs associated with late-stage interventions such as transplantation and dialysis are substantial, currently accounting for up to 3% of annual healthcare budgets in high-income countries [7–10]. Indirect costs, such as loss of productivity from early CKD-related deaths, further compound this burden. In the UK alone, projections from 2022 to 2032 estimate that CKD will contribute to 81.6 million missed workdays among diagnosed patients and an additional 11.9 million missed workdays for caregivers, reflecting the broader societal and economic impact of early mortality and morbidity associated with CKD [11]. The economic impact of CKD is especially pronounced in low- and middle-income countries where an estimated 63% of the overall global burden of CKD is concentrated [12].

Inequalities exist in CKD care, across all global settings. For example, ethnicity plays a significant role in CKD risk and outcomes, with studies reporting a higher risk of CKD progression and rate of kidney replacement therapy among Black, Hispanic and Asian populations compared with Caucasians [13]. As CKD progresses, there is also a marked deterioration in patient quality of life (QoL), with a steep decline in physical and mental QoL scores for patients with late-stage disease and those on dialysis [14], with these impacts felt more acutely within ethnic minority groups and lower-income settings. Inequalities are also documented in the prescription of CKD therapies, such as sodium-glucose cotransporter 2 inhibitors (SGLT2is), with one study reporting under-prescription amongst women, those of Black ethnicity, patients with estimated glomerular filtration rate (eGFR) <60 mL/min/1.73 m², and those of lower socioeconomic status, despite being eligible [15].

The growing burden of CKD warrants improved detection, management and care for people with this chronic, multisystem condition. Despite the availability of several comprehensive guidelines for CKD management and prevention from leading bodies such as Kidney Disease: Improving Global Outcomes (KDIGO, 2024) [16], the European Society of Cardiology (2024) [17] and the European Society of Hypertension (2023) [18], adherence remains persistently low, particularly concerning the foundational role of SGLT2is alongside renin–angiotensin system (RAS) inhibitors [2, 16, 18–20]. A recent UK primary care analysis reports that, despite 26.8% of adults with CKD meeting guideline-directed indications for SGLT2i, only 17.0% received a prescription, with <0.1% treated without coexisting diabetes [21]. Similarly, in the USA, a recent study reported SGLT2i prescribing ranging from 1.8% (CKD only) to 24.3% [CKD + type 2 diabetes (T2D) + heart failure (HF)], remaining particularly low in patients with grade 3–5 CKD [22]. This critical gap underscores the urgent need for practical, real-world examples and initiatives that effectively translate guideline recommendations into improved clinical practice, especially in early CKD management.

## CURRENT CHALLENGES

The lack of adherence to existing guidelines may be attributable to a number of existing challenges in CKD management. A major barrier is therapeutic inertia; despite strong evidence in favour of SGLT2is and RAS blockade, their uptake is slow due to uncertainty among healthcare professionals (HCPs), particularly in the treatment of CKD without diabetes [2, 20]. This hesitancy is likely to put patients at risk of disease progression and adverse outcomes [20]. Additionally, care for patients with CKD is often fragmented, with inconsistencies in applying guidelines and responsibilities in prescribing across primary and secondary care settings further impeding optimal management [23, 24].

Late initiation of therapy also reflects a general lack of screening and awareness of CKD, and the often asymptomatic nature of its presentation [23, 25–27]. Public and primary care awareness is needed to drive improvements in the early identification and management of CKD [2]. To date, research has been focused on advanced CKD, with limited emphasis on prevention and early intervention strategies. Nonetheless, several guidelines advocating for early CKD screening and management are now emerging as health systems move from treatment-based models, towards a focus on prevention [28, 29], supported by a clear cost-benefit from a public health perspective [5]. In parallel, there is also a growing body of evidence to suggest that CKD should be considered and treated under the broader umbrella of cardiovascular–kidney–metabolic (CKM) disorders, due to their interconnected mechanisms, where interventions targeting one disorder may confer benefits across the wider CKM spectrum [30, 31]. This supports an evolving paradigm that increasingly prioritizes early detection and proactive management of CKD to simultaneously improve wider long-term health implications.

With this in mind, this manuscript highlights successful initiatives in CKD management across different global regions and presents a ‘call-to-action’ to support HCPs in achieving improved CKD management globally. By bringing together eight diverse ‘Champions of Change’ initiatives, from various health systems worldwide, this paper aims to demonstrate concrete, best practice interventions across the entire CKD management pathway, from early diagnosis to treatment.

## OPPORTUNITIES FOR IMPROVEMENT

A multi-pronged approach is essential to address the challenges faced within current CKD management pathways, with several key opportunities for improvement across the patient journey.

### Improving screening and early CKD diagnosis

Firstly, early detection through systematic and opportunistic screening offers significant potential, with evidence suggesting that earlier diagnosis and intervention can delay and, in some cases, reverse progression [32]. Implementation of national policy strategies for screening and earlier intervention could substantially reduce the number of undiagnosed cases and associated complications, thereby improving long-term outcomes.

Alongside screening, enhancing education and awareness is a key pillar to improving CKD care. Increasing awareness of CKD among HCPs and patients is essential to enabling a timely diagnosis for those at risk, to maximize benefit from available and emerging therapies and improve patient outcomes [33–35]. Educating HCPs to recognize CKD as a complex, multisystem disorder, that should not be managed within siloed specialties may foster a more holistic approach to patient management and prompt recognition of early CKD indicators. Similarly, improving patient awareness and literacy through local/national campaigns and practice-based education empowers individuals to advocate for their own care, which may aid early diagnosis [2].

Clinical tools present further opportunities to streamline early diagnosis for CKD, which in turn can impact timely initiation and referral. Several validated clinical tools have been developed to optimize CKD diagnosis and risk stratification, including the Kidney Failure Risk Equation (KFRE), QKidney Calculator and the Decision Aid for Renal Therapy [36]. Integration of these tools into clinical practice can support decision-making and risk-stratification, thereby enhancing the timeliness, consistency, quality and equity of CKD care.

### Improving timely treatment initiation and adherence

Despite the availability of evidence-based guidelines, uptake of guideline-directed medical therapy (GDMT) remains suboptimal [23, 32, 33, 37]. Improving adherence to GDMT therefore presents an opportunity to help delay progression, manage comorbidities and improve QoL for patients with CKD. A particular focus should be placed on equipping primary care physicians with the tools, training and confidence necessary to initiate goal-directed therapy aligned to the current guidance. This is a global CKD priority, with the recent KDIGO Controversies Conference on Technological Advancements to Support Guideline-Informed Care aiming to identify technology-focused solutions to support greater implementation of GDMT [38]. In addition, adopting a patient-centric approach through shared decision-making in CKD management can help foster personalized care and build trust, and has been demonstrated to increase treatment satisfaction for patients with CKD [39]. Partnering with patients to co-design solutions ensures that the needs of people living with CKD are met.

### Optimizing referral to secondary care

CKD care is complex and involves multiple HCPs across different care settings [24]. Efficient collaboration and referral pathways, both between and within, primary and secondary care settings are vital to ensuring comprehensive management of CKD and its associated complications. For example, care delivered by a multidisciplinary team (MDT) has been demonstrated to slow the rate of CKD progression [40]. While effective, MDTs can be resource-intensive and create a barrier in areas lacking them. As such, there is a compelling need to upskill all clinicians who encounter patients with CKD to manage other associated conditions (i.e. primary care, cardiovascular, kidney and endocrinology experts) within a broader CKM framework. This upskilled workforce would facilitate early recognition and timely referral, especially in regions where specialist resources are scarce. As part of this, there is a need to proactively embed CKD management within primary care services, ensuring that primary care teams are equipped to address not only CKD *per se* but also common comorbidities such as hypertension, diabetes and cardiovascular disease. Such integration promotes a comprehensive, patient-centred approach, enabling more effective management and coordination across the entire care pathway.

Effective communication is also crucial. This comprises both communication throughout integrated care systems, facilitating access and sharing of information across specialties, in addition to communication with patients to ensure that they are well informed and engaged in their own care. Proper coding, documentation and structured follow-up systems are known to enhance communication and support continuity of care. In parallel, streamlined data sharing and interoperability can help support continuity of care by ensuring all HCPs involved with a patient’s care have access to their up-to-date records, particularly in CKM disorders where multiple comorbidities and specialties may be involved. This is crucial for early-stage CKD detection and management, where timely referral and escalation from primary care physicians to specialists can prevent unnecessary progression to advanced disease. Ultimately, enhancing coordination and information flow across care settings can help to improve patient outcomes and ensure continuity throughout the patient journey.

## ‘CHAMPIONS OF CHANGE’ INITIATIVES

**Figure 1:**
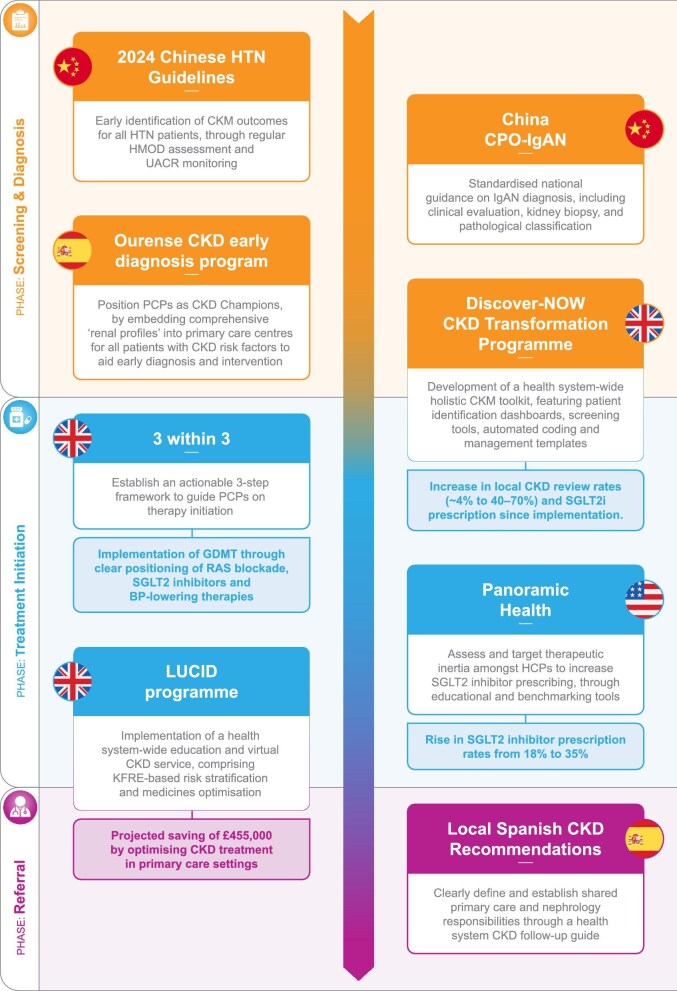
Summary of champions of change initiatives across the CKD pathway. BP, blood pressure; CPO, Clinical Practice Optimization; HMOD, hypertension-mediated organ damage; PCP, primary care physician.

Here, we present a series of initiatives from across the world that harness the abovementioned opportunities in CKD management and exemplify innovative and transformative solutions. Within these initiatives, we focus on key interventions to improve early CKD management in terms of screening and early diagnosis, treatment initiation and referral to secondary care. The eight ‘Champions of Change’ initiatives are mapped across these three stages in Fig. 1. While the initiatives are tailored to their own specific healthcare contexts, the core principles illustrate the potential of targeted interventions to significantly improve patient outcomes for CKD and wider comorbidities across the CKM spectrum. Importantly, these successes can be adapted by HCPs and system leaders to inform and implement locally relevant strategies within their own healthcare systems, advancing global efforts to prevent CKD progression and improve care for these patients.

### Screening and early CKD diagnosis with risk stratification

Many of the initiatives included interventions to facilitate earlier detection and diagnosis of CKD, through targeted recommendations, screening, diagnostic and risk-stratification tools. For example, updates to the 2024 Chinese Hypertension (HTN) Prevention and Treatment Guidelines (Supplementary data, Fig. S1) incorporated management strategies for patients with CKD and HTN, reflecting the high prevalence of HTN in China and its status as the most common modifiable risk factor driving CKD progression. The evidence-based recommendations focus on early identification of CKM outcomes, through regular hypertension-mediated organ damage assessment and urine albumin-to-creatinine ratio (UACR) monitoring, for all patients with HTN. To support this, novel automated blood pressure measurement platforms have been established in local community health centres across Shanghai, and are now being rolled out across many other provinces.

Similarly, the Clinical Practice Optimization Initiative for Chinese Patients with IgA Nephropathy (China CPO-IGAN) (Supplementary data, Fig. S2) aimed to improve clinical care for patients with immunoglobin A nephropathy (IgAN), addressing limited nationwide data on diagnosis and treatment. The project led to the development of expert consensus recommendations on IgAN management in China, which included guidance on the necessary clinical evaluation, kidney biopsy and pathological classification required for IgAN with a view to standardizing diagnosis nationally.

In Spain, the generation of local recommendations for early diagnosis and management of CKD (Supplementary data, Fig. S3) adopted a multidisciplinary approach, to target collaboration across primary care and nephrology in Madrid. A shared primary care and nephrology follow-up guide was developed to standardize diagnosis, monitoring and management, emphasizing early detection of complications and timely specialist referral [41]. Elsewhere, in the Ourense region of Spain, ongoing work is also being conducted to improve early screening and diagnosis of CKD in primary care centres (Supplementary data, Fig. S4). The Ourense initiative involves the creation of ‘renal profiles’ for all patients with CKD risk factors, to help facilitate a comprehensive analysis and monitoring of the patient’s condition, ultimately enabling early diagnosis and intervention where necessary. In particular, the team have implemented blood gas analysis as a requirement of these ‘renal profiles’, which has demonstrated successes in improving assessment and prediction of possible episodes of hyperkalaemia, aiding early detection of kidney disease.

The Discover-NOW CKD Transformation Programme in the UK (Supplementary data, Fig. S5) aimed to transform CKD care in North West London, using a multidisciplinary approach. Relevant stakeholders mapped out existing CKD pathways and identified opportunities for optimization using a bottom-up approach, to co-create, test and refine potential interventions, and implement them across primary care services. The resulting, co-designed, North West London CKD toolkit supports both patients and primary care clinicians by enhancing integrated care across primary and secondary services. This toolkit features patient identification dashboards, screening tools, automated coding guidance and management templates, exemplifying part of a system-wide approach driving proactive CKD detection and management within the broader CKM context. Since the introduction of these utilities there has been a significant improvement in metrics used to assess broad aspects of CKD care, including coding, monitoring and treatment goals, to the extent that North West London now outperforms all other sectors across England in relation to these metrics. Of note, in the period from March 2024 to March 2025, the proportion of patients whose last two eGFRs were <60 mL/min/1.73 m^2^ and who did not have a record of general practitioner–recorded CKD, reduced from 0.3% to 0.1%, well below the national average of 0.5%, and the proportion of people on the CKD Register, who have had an eGFR or uACR measurement in the last 12 months increased from 90.7% to 92.9% and 50.4% to 70.1%, respectively [42].

The LUCID programme (Supplementary data, Fig. S6) supports CKD management in the Leicester, Leicestershire, and Rutland region of the UK, by developing an integrated care system–wide education and virtual CKD service encompassing both primary and secondary care. LUCID comprises virtual clinics, medicines optimization, risk stratification using the KFRE, as well as education materials to upskill HCPs and patients in both the awareness and management of CKD. This enables HCPs to tailor management plans appropriately, ensuring that high-risk individuals receive expedited referrals and targeted interventions, leading to improved clinical outcomes and time-optimized CKD management.

These interventions collectively showcase the power of targeted policy changes, standardized diagnostic approaches and integrated, co-designed tools in significantly improving the early identification and risk stratification of CKD. They serve as compelling models for how local efforts can lead to substantial gains in delaying disease progression and enhancing patient outcomes.

### Treatment initiation

A common theme across all the ‘Champions of Change’ initiatives is the focus on timely, GDMT initiation. For example, the Chinese HTN Prevention and Treatment Guidelines (Supplementary data, Fig. S1) recommend initial therapy with angiotensin-converting enzyme (ACE) inhibitors or angiotensin II receptor blockers (ARB) for patients with CKD with proteinuria, and SGLT2is are recommended for patients with CKD with an eGFR ≥20 mL/min/1.73 m², regardless of diabetes status, to confer timely cardiorenal protection. Similarly, the China CPO-IGAN patient registry (Supplementary data, Fig. S2), which was formed to collect real-world data on management practices and adherence to therapeutic guidelines across China, identified that a high proportion of patients did not receive mycophenolate mofetil (MMF) or SGLT2is as first-line therapy. This led to the development of expert consensus recommendations that emphasize the need for early treatment initiation of RAS and SGLT2is.

In the UK, the London Kidney Network’s ‘3 within 3’ initiative (Supplementary data, Fig. S7) offers a highly practical and actionable framework aimed at primary care, specifically designed to instil confidence and facilitate early CKD treatment optimization. This intuitive approach recommends three critical steps within 3 months to significantly impact patient outcomes and the potential to save lives. The core actions aim to give primary care physicians the confidence to initiate timely treatment using the following steps, recommended at Months 1 to 3: (i) optimizing ACE inhibitor/ARB for maximum intensity RAS blockade; (ii) initiating SGLT2i, where appropriate, to leverage cardiorenal protective benefits; and (iii) initiating further antihypertensive therapy, targeting a blood pressure goal of 140/90 mmHg (or <130/80 mmHg if UACR >70 mg/mmol).

Also aiming to support primary care physicians to optimize treatment decisions, the Discover-NOW North West London CKD toolkit (Supplementary data, Fig. S5) incorporates practical materials including a review template to guide treatment initiation for patients with CKD and associated comorbidities. As part of the toolkit solutions, CKD optimization opportunities are further supported by electronic reminders within the pro-form, which are currently used to monitor and manage a range of CKM conditions, such as diabetes and hypertension. Prescription of SGLT2is increased across North West London by ∼31% between March 2024 and March 2025 [43]; however, this may also be attributed to wider educational efforts in this area, and accounts for prescribing beyond CKD alone.

The LUCID programme in the UK (Supplementary data, Fig. S6) demonstrated the substantial impact of medicines optimization and adherence to GDMT in patients with CKD. Between 1 April 2023 and 31 March 2024, a total of 590/1085 virtual patient consultations involved medicines optimization, with 62.1% of these consultations resulting in changes to patient medications in accordance with evidence-based guidelines [44]. These modifications included titration of RAS inhibitors and initiation of SGLT2is, contributing to better risk management and slowing of CKD progression [44].

In the US, Panoramic Health launched a quality improvement project specifically aimed at increasing the use of GDMT (Supplementary data, Fig. S8), with a particular focus on SGLT2is, within a value-based care patient population for Medicare patients with advanced CKD. The initiative focused on patients with comorbid heart failure, both as a key strategy for lowering avoidable admissions, but also in recognition of the broader CKM spectrum as a multi-system condition. Key insights came from comparing healthcare provider perception survey data to prescribing data, revealing that GDMT use was lower than perceived, with underlying therapeutic inertia identified as a key barrier. Providers recognized a clear opportunity for improvement, leading to a multichannel intervention that included physician-to-physician education in interactive town-hall formats.

Furthermore, the Panoramic Health initiative incorporated additional practical tools to reduce physician work burden, such as supplying providers with a script to use during patient discussions and encouraging SGLT2i indications to be included prominently at the start of clinical notes. Another key strategy that was employed was the development of performance scorecards for practices and, ultimately, individual providers. The scorecards enabled clinicians to compare their prescribing rates to their own perceptions, peers and overall network benchmarks. While this approach required a significant investment of resources, once operational, it proved to be an effective tool for focusing provider attention on the use of GDMT and enhancing adherence to best practice guidelines.

Analyses published in October 2024, assessing a cohort of patients with comorbid CKD and HF participating in a value-based Medicare programme within the network, show that as a direct result of the Panoramic Health project, SGLT2i use increased from 16.1% to 24.5%, with all-cause hospitalization (expressed as admissions per 1000 patients) reduced by 11% [45]. A broader analysis across the Panoramic network assessed 75 000 patients with an indication for SGLT2i with CKD and either T2D, proteinuria or HF, reporting a rise in SGLT2i use from 11.0% to 39.5% between November 2023 and August 2025 [46]. Overall, the programme led to improved prescribing habits for all patients with CKD, with SGLT2is now used in 46.0% of patients with CKD and diabetes; some physicians in the network report that >70.0% of eligible patients are receiving an SGLT2i (unpublished data). This initiative exemplifies a number of successful and transformative solutions that have made significant progress in enhancing provider confidence and supporting lasting prescribing behaviour change.

The diverse initiatives collectively demonstrate the critical role of targeted education, data-driven identification of barriers and the provision of practical decision-support tools in overcoming therapeutic inertia and significantly accelerating the adoption of GDMT in CKD management. The compelling outcomes, such as those from the Panoramic Health project, provide clear evidence of the profound clinical benefits achievable through concerted efforts to optimize treatment initiation, particularly in a primary care setting. Furthermore, the outcomes observed across these initiatives highlight the rapid and impactful clinical and financial benefits that can be achieved through increased GDMT, particularly with regard to prescription of SGLT2is, exemplifying how these strategic interventions can deliver system-wide improvements in a short timeframe.

### Referral to secondary care

With a focus on communication and integrated CKD care, multiple initiatives targeted their interventions on streamlining collaboration across primary and secondary care. The local Spanish consensus guidelines (Supplementary data, Fig. S3) emphasize this relationship between primary care physicians and nephrologists, clearly defining actions required at each level of care, including a recommended follow-up schedule, evaluation criteria for each visit, and the appropriate management of CKD comorbidities. Through better coordination, the guidelines enable primary care physicians to better manage earlier-stage patients, with timely referral to nephrologists [41].

Similar efforts are being championed across Spain, for example, as part of the Ourense CKD early diagnosis program (Supplementary data, Fig. S4), where a focus on improved coordination between primary care and nephrology has been established through dedicated MDTs. A ‘referents system’ has been implemented, where a group of primary care physicians, with a special interest in nephrology act as referents between their primary care colleagues and nephrologists, facilitating knowledge-sharing, timely decision-making and reducing CKD hospitalizations across the region. Through this two-way communication system, primary care physicians have become CKD experts within the Ourense healthcare system.

Within this framework of multidisciplinary collaboration, cardiorenal units have emerged across Spain as a powerful means of optimizing referrals between CKM specialists and primary care. Although models may vary according to local healthcare structure, these units are typically organized around a core team consisting of a nephrologist, a HF specialist and a team of specialized cardiorenal nurses. Depending on resource, potential added support may include primary care physicians, social workers, dietitians, pharmacists, physical therapists, vascular/dialysis surgeons, urologists, a transplantation team and palliative care specialists [47]. Funding is based on national and regional health system budget allocations, with shared cardiorenal leadership guiding protocols and patient pathways. Recent analyses of performance indicators from one cardiorenal unit, in the community of Madrid, demonstrates excellent cost-effectiveness outcomes with a significant reduction in hospitalization rate from 0.70 to 0.45 admissions per year (*P* < .02) [48]. Although comprehensive indicators demonstrating the efficacy of these interventions are difficult to define, 2023 was the first year in which a decrease in the incidence rate of patients starting renal replacement therapy was observed across Spain, falling from 152.2 per million in 2022, to 151.4 per million in 2023 [49].

The LUCID programme (Supplementary data, Fig. S6) has also led to reduced referrals to specialist clinics as well as ‘advice and guidance’ requests, through efforts to optimize care closer to home, expediting secondary care review for people at high risk or where needed. Data show that the LUCID programme helped avoid unnecessary referrals to secondary care in 84 cases (7.7%) and expedited secondary care reviews for 132 high-risk patients (12.2%), ensuring timely specialist intervention where needed [44]. Overall, the programme is projected to save approximately £1200 per virtual clinic, with a predicted annual net benefit of £455 000 by reducing clinical time spent on advice requests and increasing treatment in primary care.

These initiatives illustrate how optimized communication channels, clearly defined care pathways, and the strategic implementation of integrated care systems can bridge the traditional divide between primary and secondary care. By enabling seamless coordination, these ‘Champions of Change’ initiatives demonstrate significant improvements in patient outcomes, including reductions in hospitalizations and the potential to mitigate the progression to dialysis, alongside demonstrable economic benefits for health systems.

## LIMITATIONS

Due to the recent and ongoing implementation of the initiatives included, data to demonstrate the direct impact of the reported interventions are limited. Where data are available, they may be immature, or influenced by factors beyond the direct effects of the reported interventions, reflecting the ever-evolving landscape of CKD management. Furthermore, the availability of clinical and economic data varies across settings; in particular, obtaining reliable hospital admissions data can be challenging where access to insurance or government records is restricted, and data sharing outside value-based care contexts is often limited. Interoperability barriers, both within and between health systems also contribute to fragmented data availability, though efforts are ongoing to optimize data streamlining within some of the initiatives discussed. Finally, the described programmes operate within diverse funding and governance models, which can affect access, resources and scalability, limiting direct extrapolation to other settings.

## ‘CALL TO ACTION’ CONCLUSIONS

**Figure 2:**
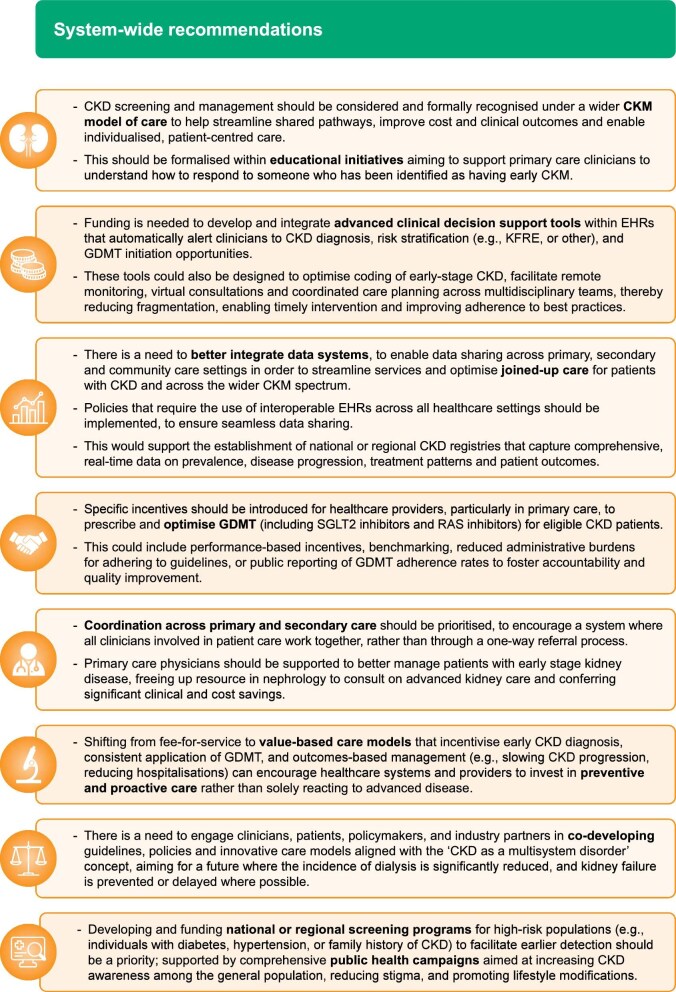
‘Call to action’ for improving CKD management: system-wide recommendations. EHR, electronic health record.

**Figure 3:**
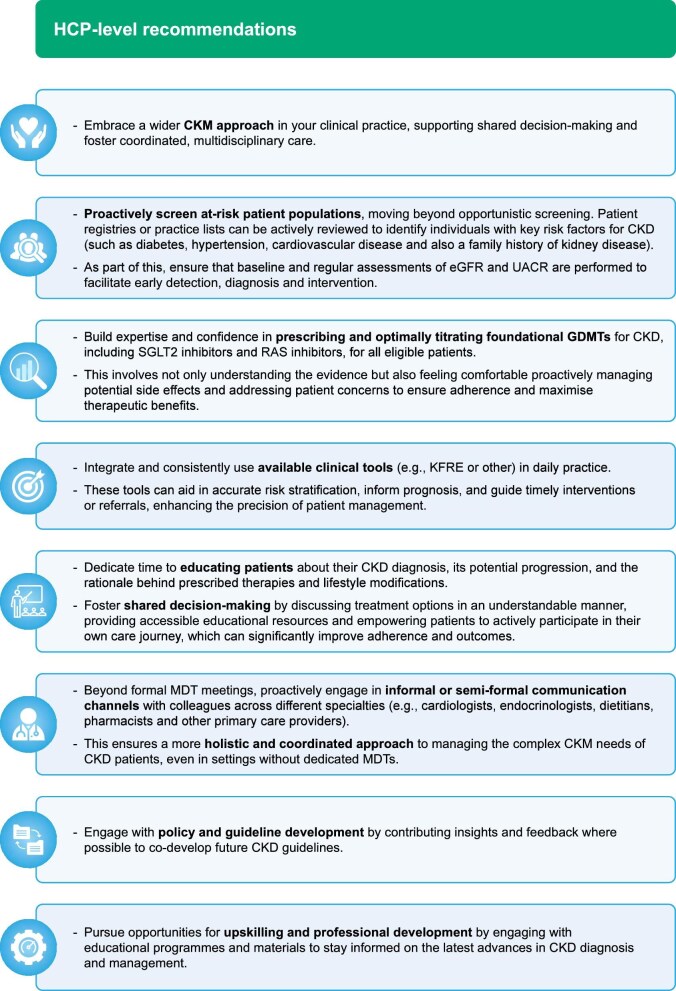
‘Call to action’ for improving CKD management: HCP-level recommendations.

Based on key learnings from the case studies presented, key recommendations are outlined at both a system level (Fig. 2) and aimed at individual HCPs (Fig. 3).

Collectively, these evidence-based recommendations, distilled from the successful ‘Champions of Change’ initiatives presented herein, offer a robust and actionable blueprint for health systems and individual clinicians worldwide. The core principles outlined here can be tailored by local system leaders, as well as at an individual HCP level, to local contexts and patient needs to drive forward targeted solutions that can help transform early CKD management. By embracing these strategies, we can collectively accelerate progress towards optimizing CKD management, thereby significantly improving patient outcomes by delaying disease progression, and addressing the wider economic impact associated with CKD on a global scale.

## Supplementary Material

sfag011_Supplemental_File

## Data Availability

No new data were generated or analysed in support of this research.
