# Supplementary material for: Transformative global models for CKD care: case studies and strategies
Source: Clin Kidney J. 2026 Jan 13;19(2):sfag011. doi: 10.1093/ckj/sfag011 (PMC12926661; doi:10.1093/ckj/sfag011)
Supplement: sfag011_Supplemental_File [file sfag011_supplemental_file.docx]

Championing Change in Early Chronic Kidney Disease Management: An International Call to Action

Supplementary Materials

James O Burton,^1^ Andrew H Frankel,^2^ Katherine Kwon,^3^ María Marqués,^4^ Gengru Jiang,^5^ Jiguang Wang,^6^ Kieran McCafferty.^7^

^1^Department of Cardiovascular Sciences, University of Leicester, UK; ^2^Imperial College Healthcare NHS Trust, London, UK; ^3^Panoramic Health, Arizona, US; ^4^Puerta de Hierro University Hospital, Madrid, Spain; ^5^Xinhua Hospital, School of Medicine, Shanghai Jiao Tong University, China; ^6^Rui Jin Hospital, Shanghai Jiao Tong University School of Medicine, Shanghai, China; ^7^Barts Health NHS Trust, London, UK

Supplementary Figure 1: Chinese Hypertension Prevention and Treatment Guidelines
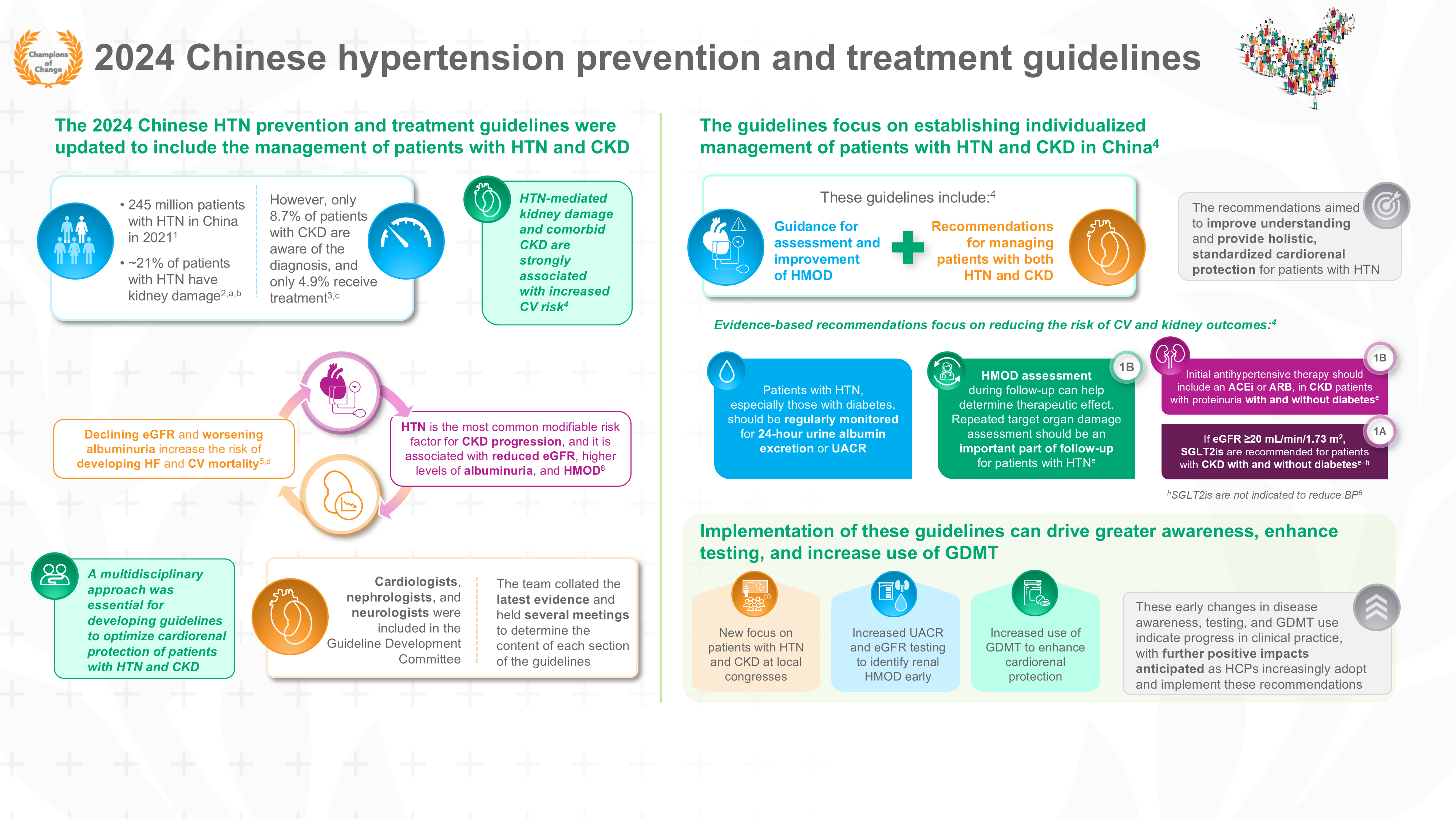


^a^According to a random, cross-sectional community epidemiological screening of 2000 patients between the ages of 18 and 75 years;^2^ ^b^Data published in 2005;^2^ ^c^Data from a cross-sectional survey conducted in 2011–2012 in Chinese people aged 45 years or older (n=11,318);^3^ ^d^Data from a collaborative meta-analysis of individual-level data from 24 cohorts in the Chronic Kidney Disease Prognosis Consortium (637,315 participants without a history of CVD), with a medium follow-up time of longer than 4 years;^5^ ^e^Class of recommendation (1 to 3) and level of evidence (A to C) shown;^4 f^Please follow the therapeutic indications and considerations of individual SGLT2is according to their approved Summary of Product Characteristics; ^g^Refer to Prescribing Information for individual eGFR cut-off points for initiation. Abbreviations: ACEi: angiotensin-converting enzyme inhibitor; ARB: angiotensin II receptor blocker; BP: blood pressure; CKD: chronic kidney disease; CV: cardiovascular; eGFR: estimated glomerular filtration rate; GDMT: guideline-directed medical therapy; HCP: healthcare professional; HF: heart failure; HMOD: hypertension-mediated organ damage; HTN: hypertension; SGLT2i: sodium–glucose co-transporter 2 inhibitor; UACR: urine albumin-creatinine ratio. 1. Bu Y, et al. China CDC Wkly 2024;6:282–288; 2. Zhang L. Chinese J Nephrol 2005;21:575–576; 3. Wang S, et al. Nephrology 2015;20:474–484; 4. Wu Z, et al. Chin J Hypertens 2024;32:603–700; 5. Matsushita K, et al. Lancet Diabetes Endocrinol 2015;3:514–525; 6. Mancia G, et al. J Hypertens 2023;41:1874–2071.

**Supplementary Figure 2: Chinese CPO-IGAN Initiative**

**
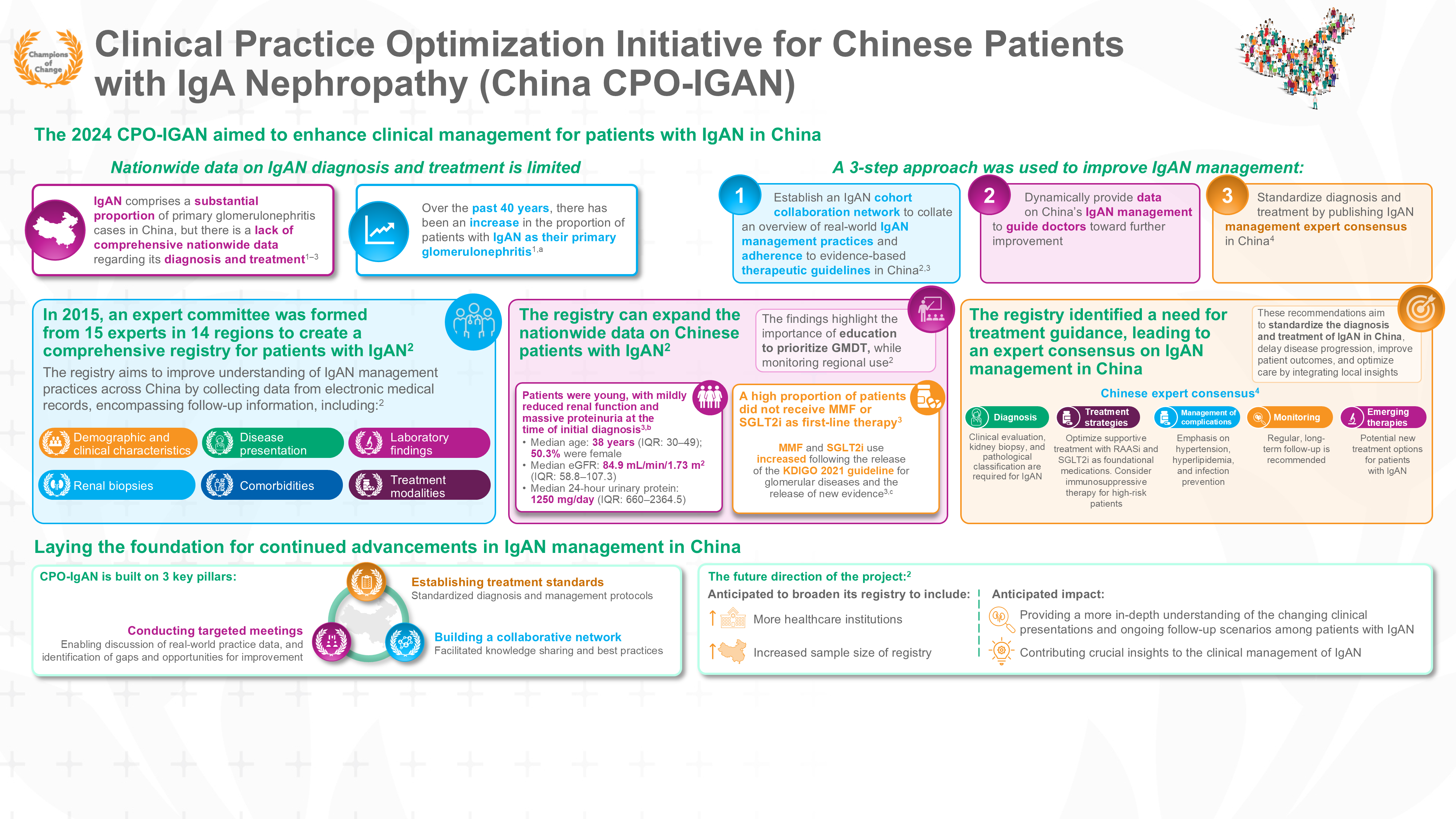
**

^a^Figures shown between 1979 and 2014 are based on a Chinese single-center study reflecting the frequencies of IgAN among individuals who have undergone renal biopsy. These results may not be applicable to the general population;^1^ ^b^A total of 2229 patients with biopsy-proven IgAN;^3^ ^c^KDIGO guidelines recommend MMF exclusively for Chinese patients as a glucocorticoid-sparing agent in those considered for glucocorticoid treatment.^5^ While DAPA-CKD and EMPA-KIDNEY studies document the reno-protective effects of SGLT2is in proteinuric CKD (confirmed by meta-analyses), KDIGO guidelines do not include SGLT2is for the management of non-diabetic IgAN.^5,6^ Abbreviations: CPO-IGAN: Clinical Practice Optimization Initiative for Chinese Patients with IgA Nephropathy; CKD: chronic kidney disease; eGFR: estimated glomerular filtration rate; GDMT: guideline-directed medical therapy; IgA: immunoglobin A; IgAN: IgA nephropathy; IQR: interquartile range; KDIGO: Kidney Disease: Improving Global Outcomes; MMF: mycophenolate mofetil; RAASi: renin-angiotensin-aldosterone system inhibitor; SGLT2i: sodium–glucose co-transporter 2 inhibitor. 1. Hou JH, et al. Kidney Dis 2018;4:10–19; 2. Jiang G, et al. Presented at ERA Congress, May 23–26, 2024. Stockholm, Sweden. Abstract no. #2389. Available at: https://academic.oup.com/ndt/article/39/Supplement_1/gfae069-1262-2389/7677418 (Accessed May 2025); 3. Lin F, et al. Presented at ASN Kidney Week, October 24–27, 2024. San Diego, CA. Abstract no. FR-PO879. Available at: https://journals.lww.com/jasn/fulltext/2024/10001/a_10_year_analysis_of_igan_management_in_china_.2402.aspx (Accessed May 2025); 4. Yue Y, et al. Chin J Kidney Dis Invest 2024;13:1–8; 5. Kidney Disease: Improving Global Outcomes (KDIGO) Glomerular Diseases Work Group. Kidney Int 2021;100:S1–S276; 6. Gleeson PJ, et al. Nephrol Dial Transplant 2023;38:2464–2473.

Supplementary Figure 3: Local Spanish Consensus Guidelines


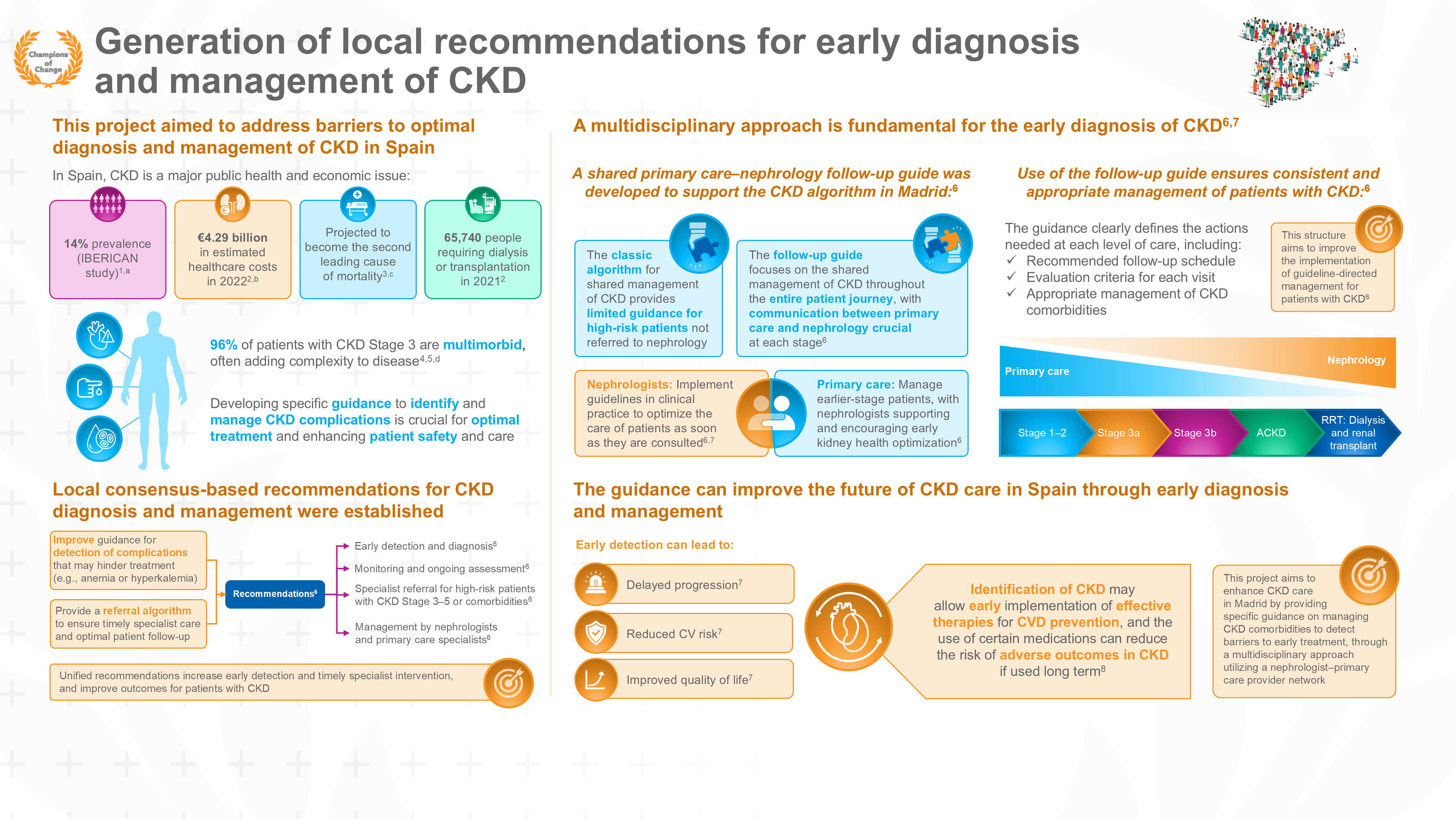


^a^The IBERICAN study is an epidemiological, multicenter, observational, prospective study, carried out in primary care centers in Spain between 2014 and 2018, with a total of 7895 patients included;^1 b^Data from Inside CKD, which uses a validated microsimulation approach to project the burden of CKD. The projection is based on a virtual population according to Spanish demographics, literature, national data registries, and clinical expert opinion;^2^ ^c^By the year 2100;^3^ ^d^Data from a prospective cohort study of 1741 people with CKD Stage 3 recruited from primary care between August 2008 and March 2010.^4^ Abbreviations: CKD: chronic kidney disease; CV: cardiovascular; CVD: cardiovascular disease; RRT: renal replacement therapy. 1. Llisterri JL, et al. Med Clin (Barc) 2021;156:157–165; 2. Navarro González JF, et al. Nefrologia 2024;44:807–817; 3. Ortiz A. Clin Kidney J 2021;15:372–387; 4. Fraser SD, et al. BMC Nephrol 2015;16:193; 5. MacRae C, et al. Br J Gen Pract 2021;71:e243–e249; 6. Working group of ERC of Community of Madrid. Recommendations on the detection, monitoring and referral criteria for Chronic Kidney Disease. Edition 2. Madrid: Department of Health. 2024; 7. ISN-KDIGO-WONCA-PCDE. CKD Early Identification and Intervention Toolkit. Commissioned and funded by AstraZeneca. Available at: https://www.theisn.org/initiatives/toolkits/ckd-early-screening-intervention/#PrimaryCare (Accessed April 2025); 8. Levin A, et al. Kidney Int 2023;103:1004–1008.

Supplementary Figure 4: Primary Care Diagnosis and Screening in Ourense, Spain


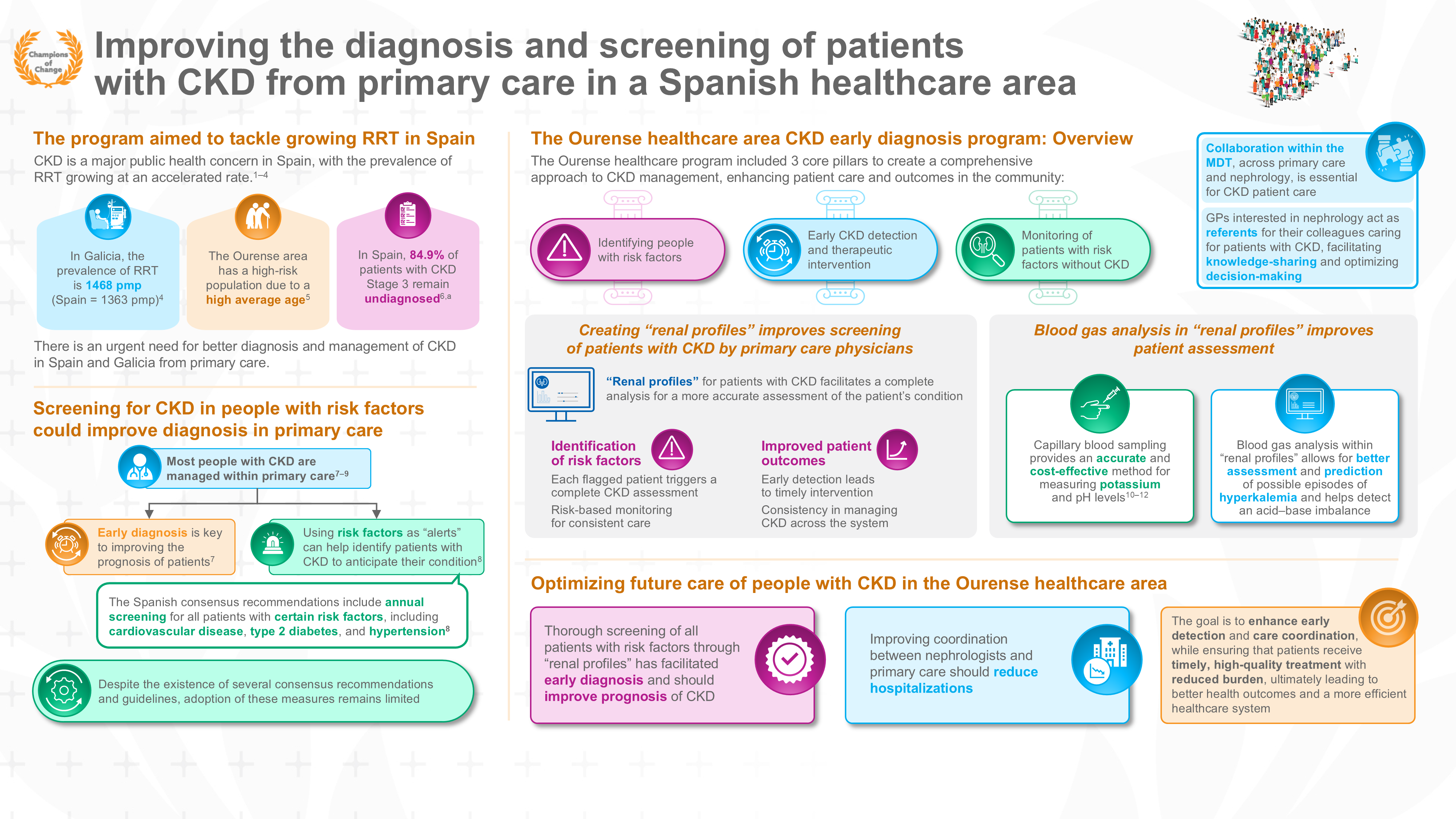


^a^Data from REVEAL-CKD, a multi-national, observational study using electronic medical records and claims data from 4 countries across 4 continents.^6^ Abbreviations: CKD: chronic kidney disease; GP: general practitioner; MDT: multidisciplinary team; pmp: per million population; RRT: renal replacement therapy. 1. Navarro González JF, et al. Nefrologia 2024;44:807–817; 2. Llisterri JL, et al. Med Clin (Barc) 2021;156:157–165; 3. Ortiz A. Clin Kidney J 2021:23;15:372–387; 4. Spanish Society of Nephrology. Chronic Kidney Disease (CKD) in Spain 2022. Available at: https://www.seden.org/documentos/la-enfermedad-renal-cronica-ercen-espana-2022 (Accessed May 2025); 5. Eurostat. Ageing Europe - statistics on population developments. Older people — where do they live? Available at: https://ec.europa.eu/eurostat/statistics-explained/index.php?title=Ageing_Europe_-_statistics_on_population_developments#Older_people_.E2.80.94_where_do_they_live.3F (Accessed April 2025); 6. Pecoits-Filho R, et al. Presented at ERA, June 15–18, 2023. Milan, Italy. Abstract no. #3667; Available at: https://academic.oup.com/ndt/article/38/Supplement_1/gfad063c_3667/7195616 (Accessed May 2025); 7. Smekal MD, et al. BMJ Open 2021;11:e046068; 8. García-Maset R, et al. Nefrologia (Engl Ed) 2022;42:233–264; 9. Otero A, et al. Nefrologia 2010;30:78–86; 10. Mahmoud H, et al. Cureus 2022;14:e23653; 11. Zavorsky GS, et al. Respir Physiol Neurobiol 2007;155:268–279; 12. Collot V, et al. Emerg Med Int 2021:7820041.

Supplementary Figure 5: UK Discover-NOW CKD Transformation Program


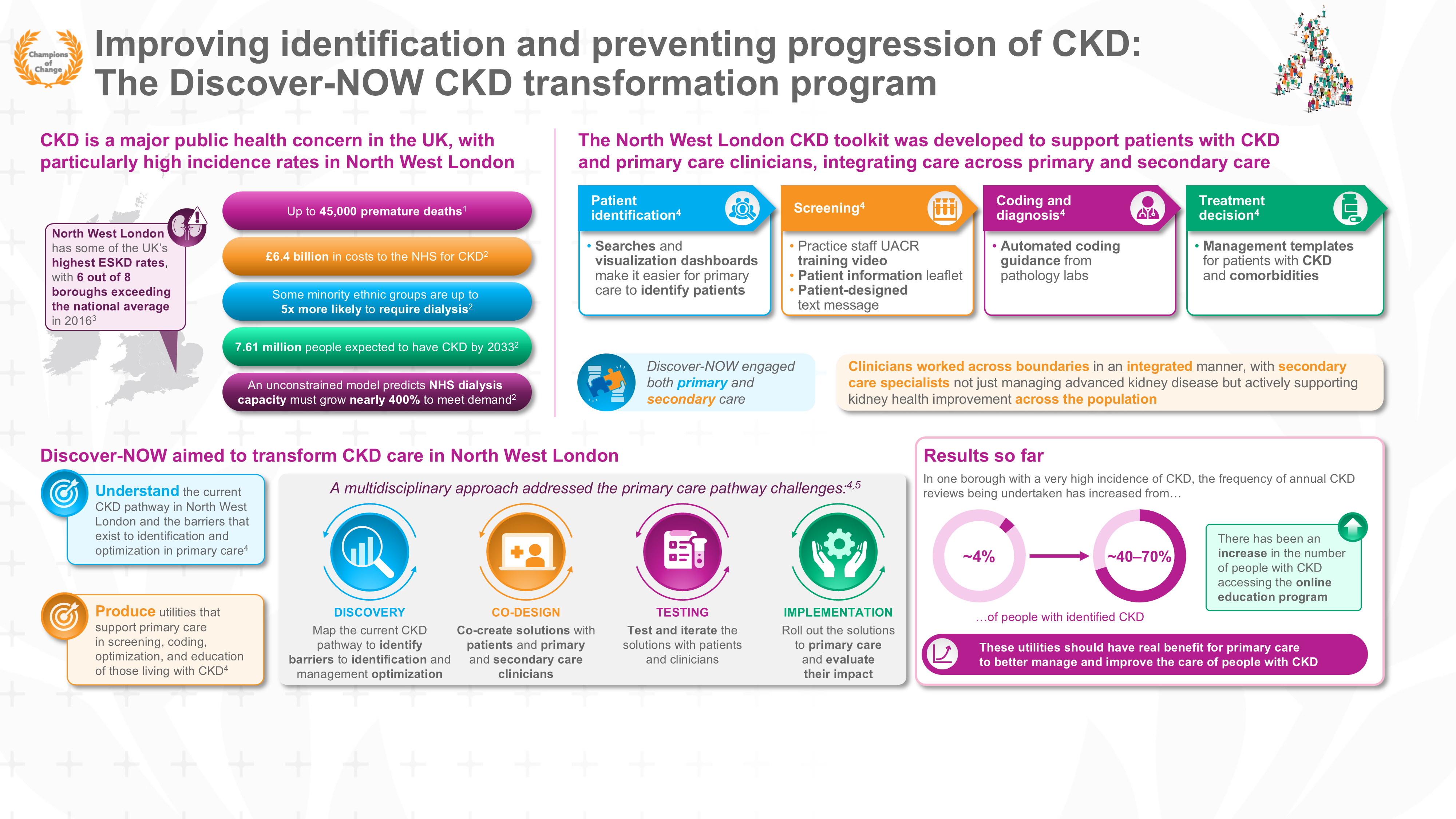


Abbreviations: CKD: chronic kidney disease; ESKD: end-stage kidney disease; NHS: National Health Service; UACR: urine albumin-creatinine ratio. 1. NHS. Chronic Kidney Disease in England: The Human and Financial Cost. Available at: <https://www.england.nhs.uk/improvement-hub/wp-content/uploads/sites/44/2017/11/Chronic-Kidney-Disease-in-England-The-Human-and-Financial-Cost.pdf> (Accessed April 2025); 2. Kidney Research UK. Kidney disease: A UK public health emergency. Available at: https://www.kidneyresearchuk.org/wp-content/uploads/2023/06/Economics-of-Kidney-Disease-full-report_accessible.pdf (Accessed April 2025); 3. Byrne C, et al. Nephron 2018;139;S1–S371; 4. London Kidney Network. Improving Identification and Preventing Progression of Chronic Kidney Disease: The DiscoverNow CKD Transformation Programme. Available at: https://londonkidneynetwork.nhs.uk/wp-content/uploads/2024/04/Project-overview.pdf (Accessed April 2025); 5. Discover-NOW. Improving identification and management of Chronic Kidney Disease. Available at: https://discover-now.co.uk/casestudy/improving-identification-and-management-of-chronic-kidney-disease/ (Accessed April 2025).

Supplementary Figure 6: UK LUCID Program


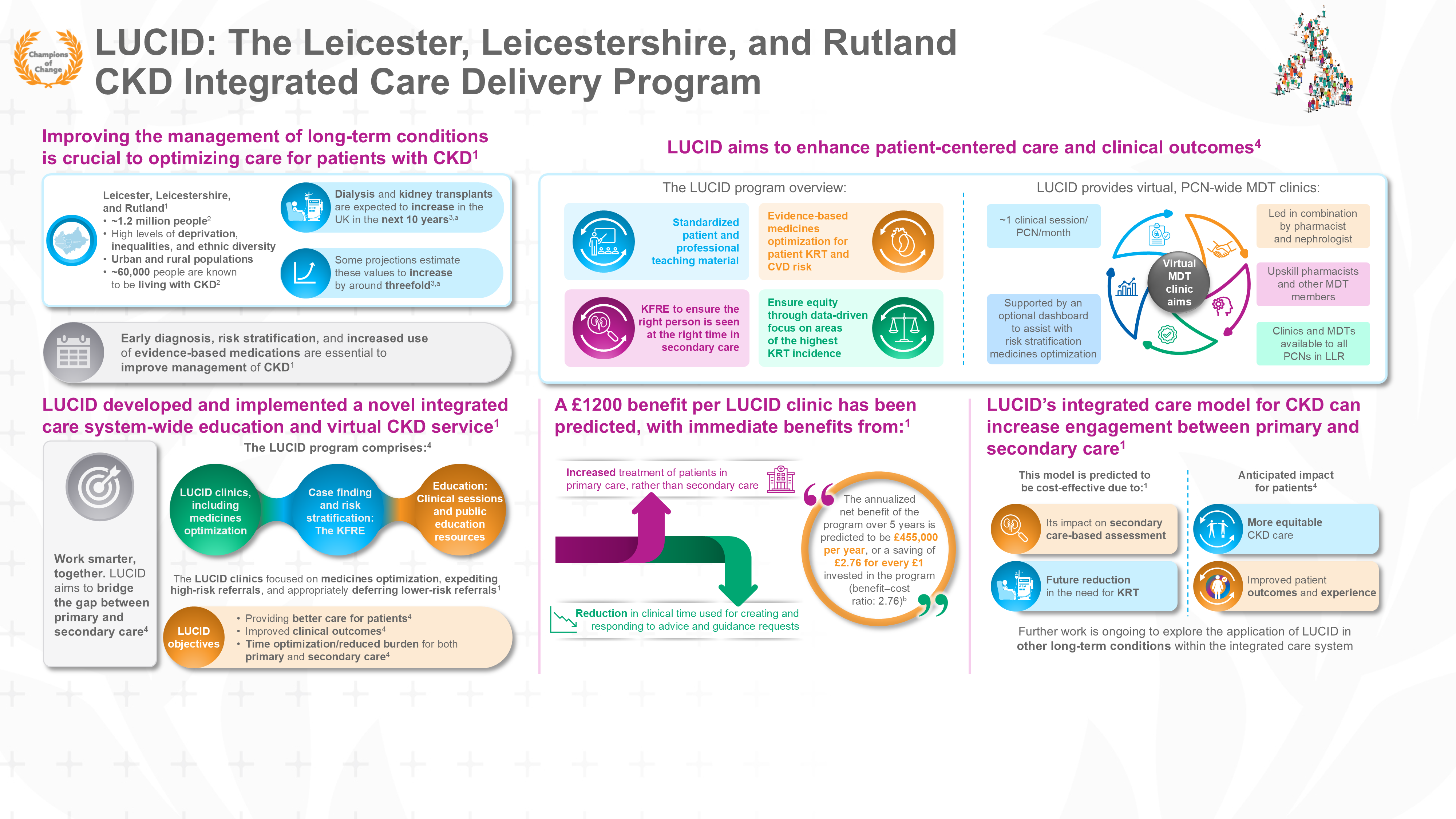


^a^Predicted benefit by external evaluation from historical activity data. These projections can incorporate any existing capacity constraints. Therefore, unconstrained projections were produced using the health economic model; ^b^Economic modeling was used to predict longer-term financial benefits in relation to reduction in hospitalization and KRT. Annualized net benefit was calculated based on participation from all PCNs. Abbreviations: CKD: chronic kidney disease; CVD: cardiovascular disease; KFRE: Kidney Failure Risk Equation; KRT: kidney replacement therapy; LLR: Leicester, Leicestershire, and Rutland; LUCID: Leicester, Leicestershire, and Rutland Chronic Kidney Disease Integrated Care

Delivery Project; MDT: multidisciplinary team; PCN: primary care network. 1. Major R, et al. Presented at UK Kidney Week, June 11–13, 2024. Edinburgh, UK. Abstract no. 299. Available at: https://www.ukkw.org/wp-content/uploads/2024/06/UKKW-2024-Abstract-book-oral-presentations.pdf (Accessed May 2025); 2. Major R and Burton J. Presented at ASN, October 24–27, 2024. San Diego, CA. Abstract no. SA-PO1119; 3. Kidney Research UK. Kidney disease is a public health emergency that threatens to overwhelm the NHS, major new report reveals. Available at:

https://www.kidneyresearchuk.org/2023/06/05/kidney-disease-is-a-public-health-emergency-that-threatens-to-overwhelm-the-nhs-major-new-report-reveals/ (Accessed April 2025); 4. NHS. Evaluation report: Leicester, Leicestershire, and Rutland Chronic Kidney Disease Integrated Care Delivery Program (LUCID). Available on request at: https://leicesterleicestershireandrutland.icb.nhs.uk/pilot-programme-for-patients-with-chronic-kidney-disease-will-be-expanded-following-its-success/ (Accessed April 2025).

Supplementary Figure 7: UK 3 Within 3 Framework
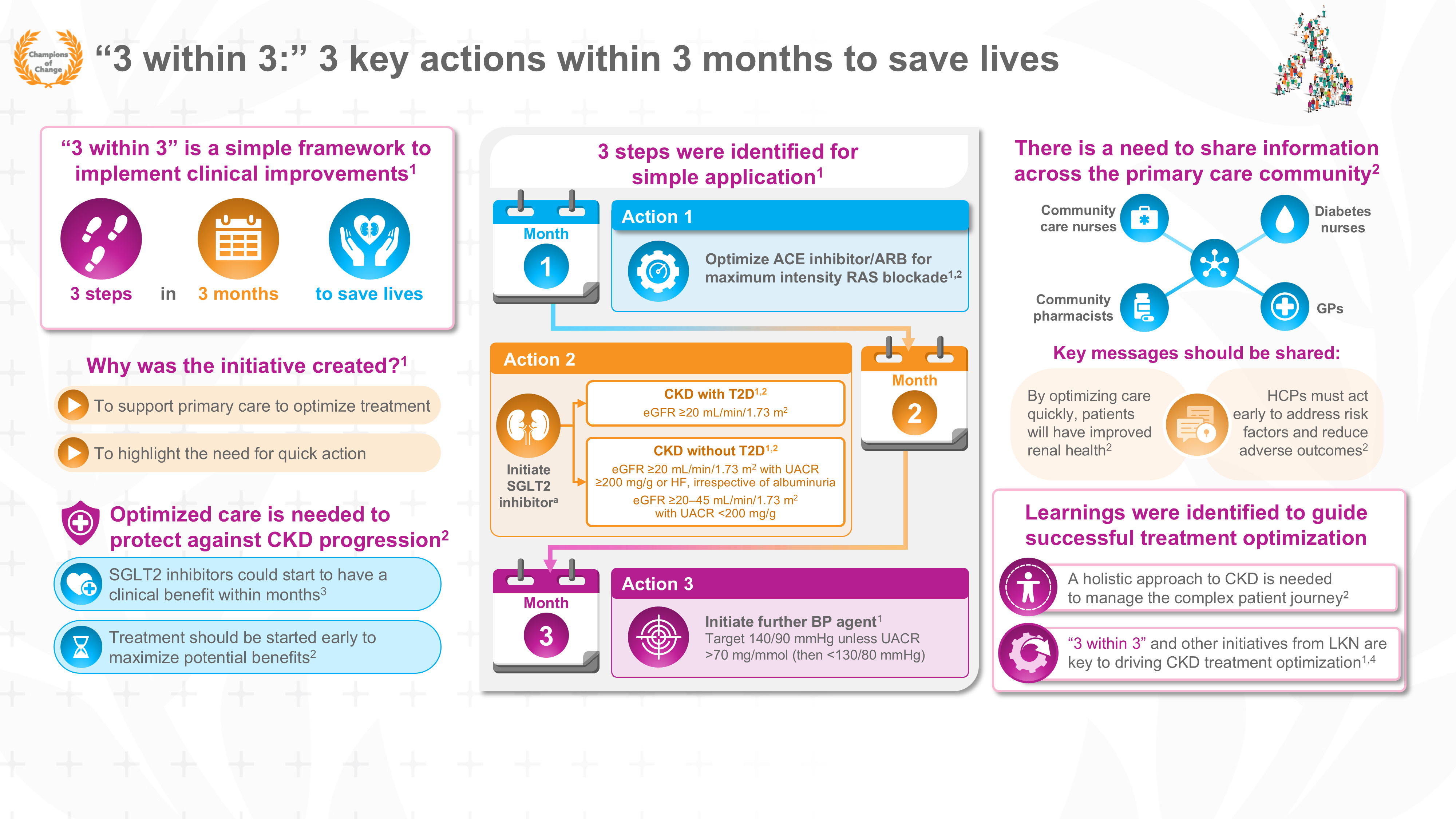


^a^Refer to individual SGLT2 inhibitor prescribing information for eGFR cut-off points for initiation. Abbreviations: ACE: angiotensin-converting enzyme; ARB: angiotensin II receptor blocker; BP: blood pressure; CKD: chronic kidney disease; eGFR: estimated glomerular filtration rate; GP: general practitioner; HCP: healthcare professional; HF: heart failure; LKN: London Kidney Network; RAS: renin-angiotensin system; SGLT2: sodium–glucose co-transporter 2; T2D: Type 2 diabetes; UACR: urine albumin-creatinine ratio. 1. London Kidney Network. CKD in Primary Care: new approaches to reduce inequalities and save lives: LKN CKD Early Identification and Optimisation Pathways (3 in 3). Available at: https://londonkidneynetwork.nhs.uk/wp-content/uploads/2024/09/LKN-CKD-Early-Identification-Pathway-19.9.24-final-v2.3.pdf (Accessed April 2025); 2. Kidney Disease: Improving Global Outcomes (KDIGO) CKD Work Group. Kidney Int 2024;105(Suppl. 4S):S117–S314; 3. Madero M, et al. Kidney Med 2024;6:100851; 4. London Kidney Network. Workstreams and Advisory Groups. Available at: https://londonkidneynetwork.nhs.uk/clinical-workstreams/ (Accessed April 2025).

Supplementary Figure 8: US Panoramic Health GDMT Optimisation Program


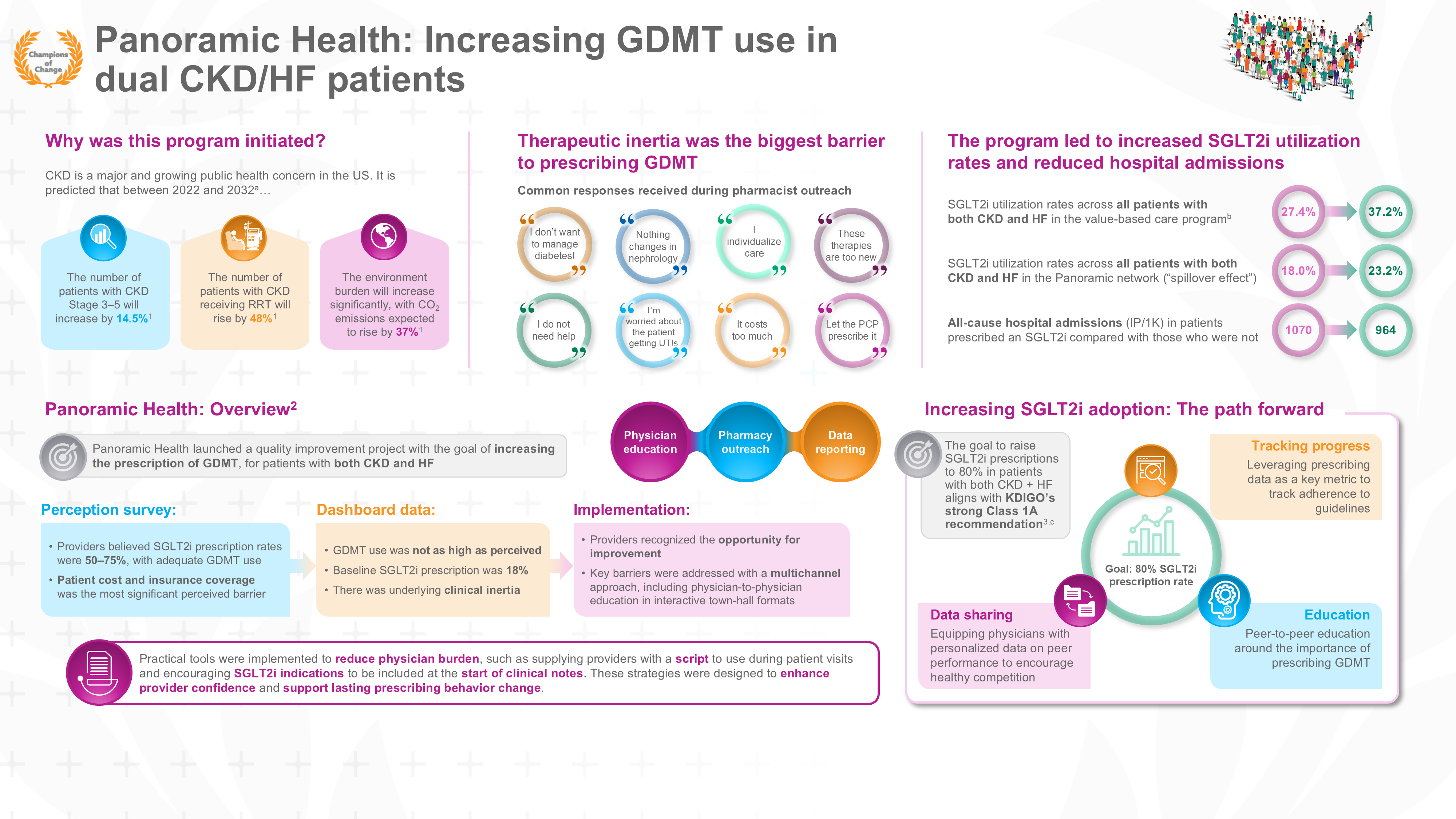


^a^Based on IMPACT CKD, a patient-level simulation model that simulates the natural history of CKD and forecasts the 10-year impact of CKD;^1^ ^b^Patients in the value-based care program, which equates to ~5% of the patients in the Panoramic network; ^c^KDIGO recommendations and classification: Grading of recommendation (1 or 2) and level of evidence (A to D). Strength of recommendation: 1 = strong, 2 = weak; Quality of evidence: A = High, B =Moderate, C = Low, D = Very low.^3^ Abbreviations: CKD: chronic kidney disease; GDMT: guideline-directed medical therapy; HF: heart failure; IP/1K: inpatient per 1000 patients; KDIGO: Kidney Disease: Improving Global Outcomes; PCP: primary care physician; RRT: renal replacement therapy; SGLT2i: sodium–glucose co-transporter 2 inhibitor; UTI: urinary tract infection. 1. Priest S, et al. Presented at ASN Kidney Week, November 2–5, 2023. Philadelphia, PA. Abstract no. FR-PO941. Available at: https://journals.lww.com/jasn/citation/2023/11001/impact_ckd__projecting_the_growing_environmental.2356.aspx (Accessed May 2025); 2. Kwon K, et al. Presented at ASN Kidney Week, October 22–27, 2024. San Diego, CA. Abstract no. TH-PO1045. Available at: https://journals.lww.com/jasn/fulltext/2024/10001/increasing_use_of_sglt2_inhibitors_in_patients.1368.aspx (Accessed May 2025); 3. Kidney Disease: Improving Global Outcomes (KDIGO). Kidney Int 2024;105:S117–S314.
